# In vitro bioactivity, cytotoxicity, and gene silencing of graphene oxide as a Bcl-2 siRNA carrier in osteosarcoma cells with in vivo inflammatory response

**DOI:** 10.1038/s41598-026-48934-0

**Published:** 2026-04-17

**Authors:** Shanmuga Sundar Saravanabhavan, Zsolt Sarang, Nivetha Marimuthu, Sowri Victor Varun Raju, Veeranoot Nissapatorn, Consolato M. Sergi

**Affiliations:** 1 Department of Biotechnology, Aarupadai Veedu Institute of Technology, VMRF (DU), Chennai Campus, Paiyanoor, Chennai, Tamil Nadu India; 2 Department of Biochemistry and Molecular Biology, Faculty of Medicine, University of Debrecen, Debrecen, Hungary; 3 Futuristic Science Research Center, School of Science, Southeast Asia water Team (SEA Water Team) and World Union for Herbal Drug Discovery (WUHeDD), and Research Excellence center for Innovation and Health Products (RECIHP), Walailak University, Nakhon Si Thammarat, 80160 Thailand; 4 Department of Laboratory Medicine and Pathology, University of Alberta, Edmonton, AB Canada

**Keywords:** Graphene oxide (GO), *bcl-2*, Inflammation, *siRNA*, Biocompatibility, Biochemistry, Biotechnology, Cancer, Drug discovery

## Abstract

Research on using graphene oxide (GO) as a potential carrier for small interfering RNA (siRNA) in the treatment of osteosarcoma is currently under intense investigation. Here, we focus on evaluating the GO as a carrier of *bcl-2* siRNA in osteosarcoma cell lines and assessing its bioactivity, and the inflammatory response of GO in non-tumour models in vivo. GO was synthesized using a modified Hummer’s method, and the physiochemical characterization confirmed its formation. The results of the MTT (3-(4,5-dimethyl-2-thiazolyl)-2,5-diphenyl-2 H-tetrazolium bromide) experiment confirmed comparable dose-dependent inhibition of viability, while staining of MG-63 cells through PI (propidium iodide) showed comparatively higher viability and concluded that GO alone cannot efficiently induce widespread apoptosis yet boosts cytotoxicity through collaboration with siRNA. GO exhibited an early cytokine expression peak as compared with the control group, with a peak at 7–14 days and then a gradual decline at 21–28 days, indicating an initial adaptive immune response following through adaptation, supporting general biocompatibility but not disease-specific efficacy. The siRNA in vitro assay exhibited a high burst release from the GO sheets and covered approximately 35–40% at 30 h. The biomaterial was observed to be bioactive in vitro and may facilitate osteointegration if applied in vivo. Despite GO’s hydrophilic nature with smooth edges and high oxygen level with a generally low toxic tendency, we still observed a certain notable level of toxicity. If the quantity of GO were increased, then the number of neutrophils (F4/80^low^, Ly6C^high^) got depleted and was less as compared with those of the control groups and could expose patients to a risk of secondary infection. The qPCR expression study of a number of cytokines (IL-6, TNF-α, TGF-β, IL-1α, IL-10, and MIP-1β) at diversified time points reiterated findings of protein expression. However, modulation of bcl-2 expression using GO as a carrier could contribute toward innovative approaches for osteosarcoma, pending validation in tumour-relevant models.

**Supplementary Information:**

The online version contains supplementary material available at 10.1038/s41598-026-48934-0.

## Introduction

Graphene, often referred to as the “wonder material,” is a two-dimensional carbon allotrope that is sp^2^ hybridized and arranged in a honeycomb lattice. It has a unique optical property: it is transparent and absorbs a large fraction of infrared and ultraviolet radiation. It can also be bent, stretched, and folded without breaking. These properties make them ideal for flexible and wearable devices, such as sensors and membranes. GO, fluorographene, reduced graphene oxide (rGO), graphyne, graphone, graphene-doped derivatives, graphene, and graphdiyne are some of their derivatives^1–4^. Biomedical researchers frequently research graphene due to its large interfacial area, which makes it an ideal choice for high drug loading. Similarly, delocalized p-electrons enhance the solubility of drug molecules when applied in vivo. In addition, when modified, graphene’s hydrophilic-lipophilic nature makes it a versatile candidate for delivering drugs to the interior of a cell by interacting with the cell membranes^1,5–8^. Numerous investigations have been conducted to explore the use of graphene as a carrier for DNA, RNA transfer, medication delivery, tissue engineering, and cell culture in therapeutic diagnostics. Zhao et al.^9^ reported a drug-loading efficiency of graphene to be around 200%, which is comparatively higher than that of many existing nanocarriers^9,10^. Another factor that makes graphene-based materials fascinating is their ability to deliver drugs at a higher rate with ease and on target. These characteristics make graphene and its derivatives ideal candidates as biomaterials.

Among children aged 1–14 years, pediatric cancer continues to be the primary cause of disease-related mortality^11,12^. While many risk factors, such as pesticide exposure, high-dose radiation, and certain genetic disorders, have been definitively established, the cause of most occurrences is still unknown. Osteosarcoma is a malignant neoplasm affecting the skeletal system with a very complex pathogenesis and endocrinic influences^13–22^. It is widely acknowledged that it is the predominant form of cancer after the Ewing sarcoma or primitive neuroectodermal tumour. The prognosis is concerning and is not maintained by administering conventional treatment regimens. Significant incidences of recurrence and metastasis frequently coexist with this malignant tumour. Chemotherapy often becomes ineffective due to the emergence of multidrug resistance, even though the process by which chemotherapy resistance develops remains fascinating and involves complex pathways. Researchers used GO to charge photosensitive agents aimed at treating anti-tumour drugs, such as SN-38 and hypocrellin, while also investigating the device’s photodynamic therapies and chemotherapy capacity^23,24^. A study also showed that graphene quantum dots had higher amounts of water than traditional photosensitizers, such as porphyrins, due to the multi-state sensitization process. In electrostatic interactions between a negative GO charge and a positive methylene charge, Zhang et al.^25^ synthesized methylene blue-loaded nanoGO^25^. Methylene blue is used in photodynamic therapy as a photosensitizer for high oxygen quantum yield. In acidic conditions, the accelerated release of methylene blue is attributed to the reduction of electrostatic forces between nano graphic oxides and methylene blue, resulting in the reduction of electrostatic power between these two entities. This mechanism is pH-dependent, as revealed by the accelerated release of methylene blue. Graphene is applied in a hybrid set of chemotherapy and phototherapy. It is a technique for decreased drug and multidrug resistance due to its large interfacial area and high drug-loading capability. They loaded doxorubicin and irinotecan to graphene and researched the combination of chemotherapy and phototherapy. A combination of photo dynamics and phototherapy can be given by loading a photosensitizer on the GO surface^25^. Another study reported a direct method for loading hydrophobic materials onto various commercially available nano- and micro-polymeric particles by modifying their surface through layer-by-layer (LbL) self-assembly^26^. Their study have a wide range of applications for administering drugs to the body^26^. GO signals can be used as a Raman probe for imaging cells and tissues^27^. The low Raman GO signal can be optimized using surface-enhanced Raman dispersal markers. The hybrid GO prepared helps improve the nanoparticles’ Raman surface scattering. Mittal et al. (2018) were among the first scientists to use Doxorubicin (DOX), an anti-cancer drug, in an unmodified, pristine graphene oxide (GO). GO-primed medicine was easily combined with GO and DOX solutions; 0.5 h was allowed to suction, and afterward, it was stirred overnight at atmospheric pressure^28^. Centrifugation eliminated unbound DOX molecules, and two of the most robust types accompanied GO-DOX formation: fluorescent dyes and electrochemical experiments. At 590 nm with oscillation at 480 nm, free DOX displayed high fluorescence emissions^24^. However, GO-DOX demonstrated clear quenching, owing to the electron transfer picture at the GO-DOX interface. GO-DOX displays improved CV curves compared to free DOX and free GO in the gradual voltammetry measurement. The “granulated” surface of GO revealed that CPT was effectively adsorbed into the GO layers, causing swelling and increasing its height profile. GO nanosheet layers are held together by the stacking of µ-α and hydrogen interlayer links^29,30^. As drug molecules, such as DOX, were added, the hydrogen connection between GO nanosheets was stopped, and the hydrogen connection between GO-DOX and DOX-DOX was substituted. Besides, GO-DOX and DOX-DOX Ţ-β stacking have also disrupted and displaced the long-range contact between GO nanosheet layers^24^. The analysis also explicitly shows that the drug molecules, rather than the multi-lag stack, inhabit empty spaces on the GO surface.

**Fig. 1:**
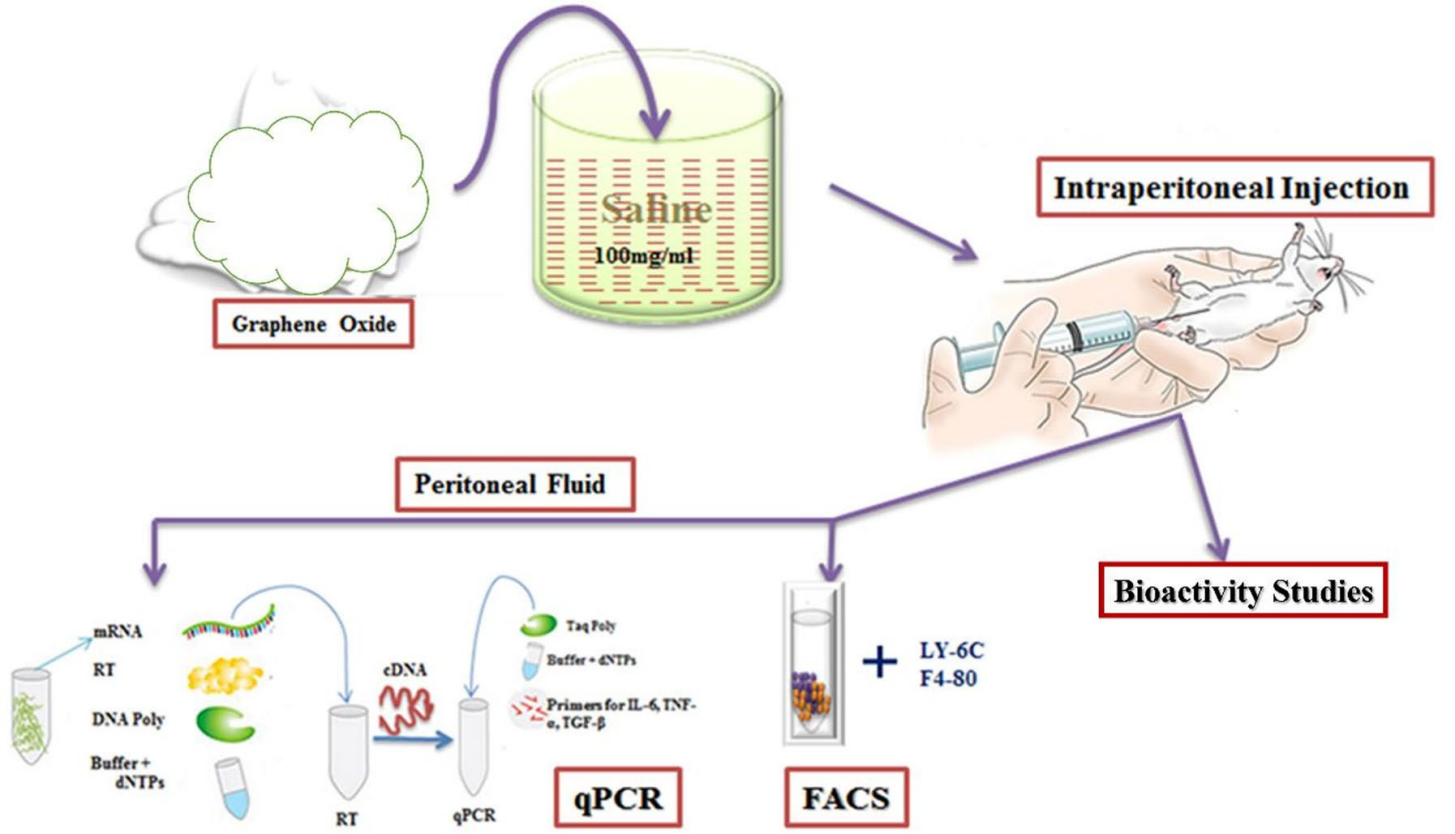
Schematic representation of the work carried out in the present study where graphene oxide dispersed in saline was administered to mice via intraperitoneal injection. Peritoneal fluid was subsequently collected and analyzed using qPCR and FACS to evaluate gene expression and immune cell populations, providing an overall assessment of bioactivity.

Graphene-based materials are extensively used in dental implants. An effective measure of mechanical failure avoidance after implantation is a key feature of dental implants^31^. Analysis has shown that silicate titanium plates are more rigid than other carbon-graphite polymers and have excellent adhesion with a higher bond resistance. Our present study evaluates GO as a potential siRNA carrier for inhibiting bcl-2 in osteosarcoma cells, focusing on in vitro cytotoxic activity and in vivo biocompatibility via inflammatory response on non-tumour mice models (Fig. 1). While in vitro assays test gene silencing efficacy, the non-tumour intraperitoneal model assesses general systemic effects, acknowledging limitations for direct disease translation. GO is synthesized using Hummers’ method, with cytotoxicity, bioactivity, and inflammatory dynamics examined in vitro and in vivo.

## Methods

### Materials

GO synthesis was carried out using graphite powder (Sigma product number: 282863), sulfuric acid (H₂SO₄), phosphoric acid (H₃PO₄), potassium permanganate (KMnO₄), hydrogen peroxide (H₂O₂), distilled water, and hydrochloric acid (HCl) procured from Sisco Research Laboratories (SRL) Pvt. Ltd. . The MTT and ROS assays were carried out as per the manufacturer’s protocol (Roche, Germany). The bioactivity is tested using SEM for surface morphology, EDS is used to show the elemental composition, AFM for surface topography and roughness. The siRNA for the study was obtained from Thermo Fisher Scientific, India (AM16708). Reagents used for FACS analysis included Alexa Fluor 488-conjugated anti-F4/80 antibody (MF48020, Invitrogen, Thermo Fisher Scientific, USA), Ly6C PerCP-Cy5.5 (128012, BioLegend, San Diego, CA, USA). All studies involving mice were carried out in accordance with the University of Debrecen (DEMAB) Ethical Committee for Animal Care (Ethics Approval #7/2016/DEMÁB at the University of Debrecen, Debrecen, Hungary)^32^. The study was conducted according to the guidelines of the Declaration of Helsinki and approved by the Institutional Ethics Committee of University Debrecen under the protocol code 28/2017/DEMÁB as per ARRIVE guidelines.

### Preparation of graphene oxide (GO)

**Fig. 2:**
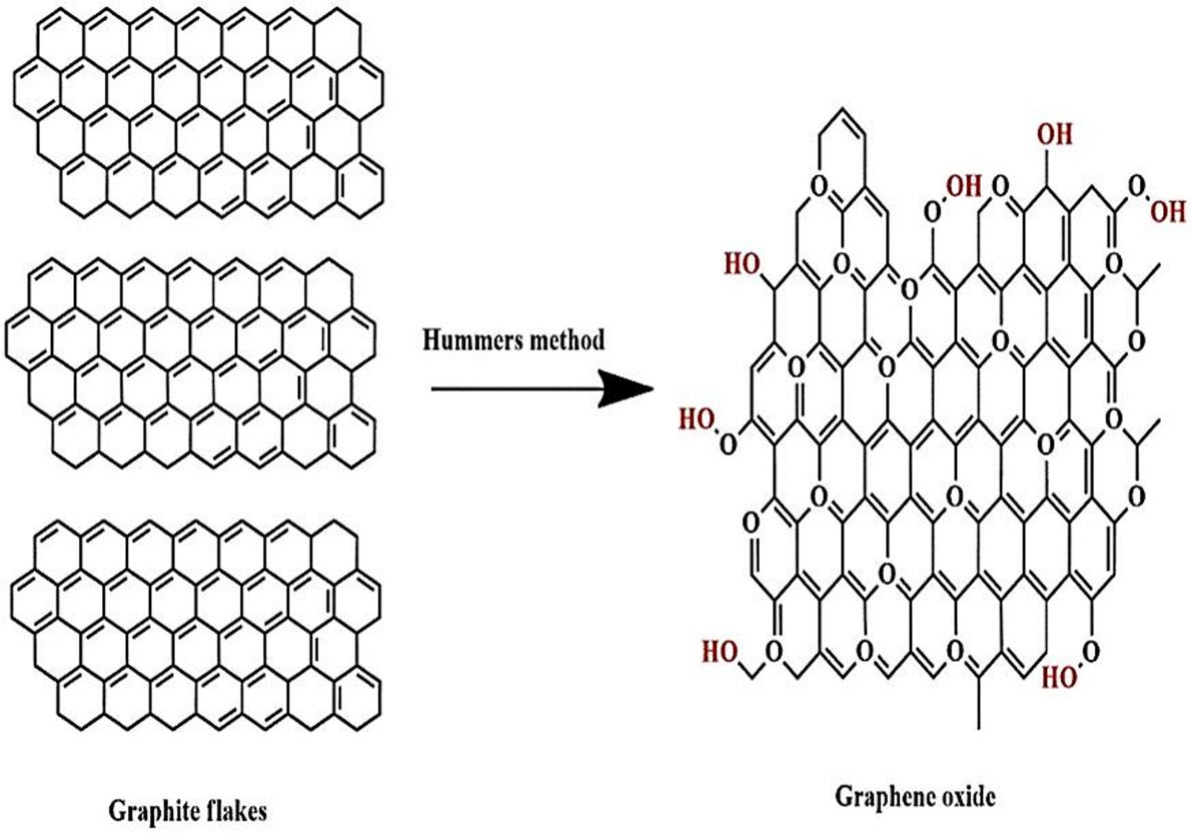
Synthesis of Graphene oxide from graphite flakes using hummers method.

A modified Hummers technique was used to synthesize GO from pure graphite powder (Fig. 2). In a typical procedure, in a 9:1 weight ratio, 27 mL sulfuric acid (H_2_SO_4_) and 3 mL phosphoric acid (H_3_PO_4_) were added and stirred together. The solution was then treated with 0.225 g of graphite powder while being stirred, followed by 1.32 g of potassium permanganate (KMnO_4_). The solution was swirled for 6 h until it turned dark green. To remove excess KMnO_4_, 0.675 mL of hydrogen peroxide (H_2_O_2_) was gently added to the solution and stirred for 10 min. The solution was allowed to cool due to the exothermic process. After the mixture was cooled, 10 mL of hydrochloric acid (HCl) and 30 mL of deionized water (DIW) were added and agitated at 5000 rpm for 7 min. After decanting the supernatant, the residuals were washed thrice with HCl and DIW. The cleaned GO mixture was oven-dried at 90 °C for 24 h to get the synthesized GO powder. The synthesized GO was characterized using SEM, TEM, XPS and XRD for their physio chemical properties.

### Study on the GO

GO synthesized through the Hummers method was used in the present study to evaluate its effectiveness as a carrier of siRNA in vitro and to assess its inflammatory response both in vivo and in vitro in mouse models.

### The siRNA release kinetics with different concentrations and pH values

The siRNA is incorporated with GO at different concentrations and pH values to study the drug release kinetics. Complexation of siRNA with graphene oxide (GO) was performed by mixing GO (1 mg/mL) with siRNA (20–100 µM) in phosphate-buffered saline (PBS, pH 7) followed by 1 h incubation at room temperature. The interaction between GO and siRNA occurred primarily through π–π stacking and electrostatic attraction. For UV–Visible analysis, the baseline absorbance of GO alone was subtracted to account for background interference. The pH values used for GO incorporated with siRNA are 3,5 and 7. The solutions with different concentrations are then treated with a phosphate buffer solution. This was followed by recording the absorbance of these solutions at 260 nm using a Nanodrop (Thermo Scientific Nanodrop 2000 Spectrophotometer) at a 30-minute time gap. The release kinetics were analyzed using the Peppas model by fitting the data to the corresponding mathematical equation^33^.

### In vitro bioactivity studies

The in vitro bioactivity of GO formulations was evaluated following the procedures adapted from our previous studies. Briefly, GO were immersed in simulated body fluid (SBF; Kokubo’s solution, pH 7.25) for 0, 7, 14 and 21 days at 37 °C to assess formation of apatite on the surface of GO. After incubation, the samples were removed from SBF, rinsed with deionized water, dried, at room temperature. The surface morphology was then examined using a SEC High-Resolution Scanning Electron Microscope (Model No: SNE-4500 M Plus(B)); EDS – Bruker XFlash^®^ 600 Mini silicon drift detector) and AFM to confirm the apatite growth or bioactivity over the membrane surface.

### In vitro biocompatibility analysis

The prepared sample’s in vitro or non-immunogenic analysis is important to check its biocompatibility. The MTT and ROS assays were performed to check the toxicity of GO NPs with the osteoblast cell line (MC3T3-E1, 7F2, 2E8) using a standard protocol adapted from our previous studies. MTT assay was carried out after incubation at 48 h to evaluate cell viability using UV Visible Spectroscopy at 528 nm^32^. Briefly, Saos-2 (catalog HTB-85) and MG-63 (catalog CRL-1427), both originally sourced from the American Type Culture Collection (ATCC, Manassas, VA, USA). For the biocompatibility experiments, MC3T3-E1 (ECACC 99072810), 7F2 (ATCC CRL-12557), and 2E8, which came from ATCC. All cell lines were maintained under standard incubator conditions (37 °C with 5% CO₂) using the media recommended by the suppliers—McCoy’s 5 A plus 15% FBS for Saos-2, EMEM plus 10% FBS for MG-63, and α-MEM plus 10% FBS for the mouse lines. We regularly checked the cultures for mycoplasma and kept passage numbers low to ensure the cells behaved consistently.

### Blotting studies

Saos-2 and MG-63 cells were placed in 6-well plates (2 × 10⁵ cells/well) and given time to settle for a night. For 48 h, GO-complexed scrambled and siRNA bcl-2-targeting were added to cells in volumes of 20, 40, 60, 80, or 100 µL. Upon the completion of treatment, the monolayers underwent two washing cycles with cold PBS and then were lysed with RIPA buffer (50 mM Tris-HCl, pH 7.5, 150 mM NaCl, 1% NP-40, 0.5% sodium deoxycholate, 0.1% SDS), and then a protease inhibitor cocktail was added to it. Centrifugation was performed at 14,000 × g, 15 min, 4 °C for clarification to lysate the cells, and protein content was determined by BCA assay. In Laemmli buffer, equal volume (25–30 µg) of protein was denatured, separated by 12% SDS-PAGE gels, using wet transfer (100 V, 90 min, 4 °C) and transferred to PVDF membrane. Membranes were treated with 5% non-fat milk for 1 h at room temperature in TBST (TBS + 0.1% Tween-20) and were then incubated with primary antibodies: anti-bcl-2 (1:800) and anti-β-actin (1:3000) in blocking buffer overnight at 4 °C. Following washing (TBST, 3 × 10 min), HRP-conjugated secondary antibody (1:5000) was applied for 1 h. Bands were made visible on a chemiluminescence imager.

### In vivo studies

ARRIVE compliance was ensured by incorporating details of randomization, blinding, and predefined humane endpoints (e.g., weight loss < 20%) in the experimental design. The study was carried out using 6-week-old mice, with parallel control groups: (i) untreated/negative control (healthy, un injected mice, *n* = 6); and (ii) positive control (LPS-injected, 20 µg/mL, *n* = 6). The planned sample size was determined by the G*Power program to be 6–6 mice/time point in the two experimental groups, providing a sufficient sample size for unpaired t-tests. For the selection of the animals, no inclusion or exclusion criteria were made. Animals from each cage were randomly allocated to the control or treated groups, but no blinding was used. Mice were monitored for humane endpoints (e.g., do not feed, > 20% weight loss) as per ARRIVE compliance. Briefly, the in vivo inflammatory response of GO was evaluated by injecting 0.5 mL and 1 mL of 10 mg/mL of GO in the peritoneal cavity of mice, followed by observation at different periods (0th day (wild strain), 7th day, 14th day, 21st day, and 28th day). After this, the peritoneal fluid was extracted from mice with and without treatment for further analysis.

#### Protein expression (FACS analysis)

The cells were extracted from peritoneal fluid, and the RNA isolated from them was used for further studies. LY-6 C and F4-80 stains were used to check for inflammation. The cells were centrifuged for purity. 1 mL of ice-cold buffer was suspended with 2 × 10^6^ cells, as the cell number affects staining quality. The cell suspension was pre-coated in tubes with 2% bovine serum albumin (BSA) in PBS. The cell suspension was added dropwise to 9 mL of 70% ethanol in a 15-mL centrifuge tube during slow vortex, followed by centrifugation of the cells at 200 rpm for 10 min at 40 °C. The obtained pellet was suspended in 3 mL of cold PBS and transferred to a tube (Falcon^®^ 12 × 75 mm (Cat. No^35^. 2054) polystyrene tubes) for staining. The cells were washed with cold PBS and resuspended in 400 µL of Ly6C/F480 staining solution.

#### Gene expression (qPCR)

The peritoneal fluid was lysed using an RNA extraction kit, and the purity was quantified using a nanodrop spectrophotometer (Thermo Scientific 2000 series). The isolated RNA is converted into complementary DNA (cDNA) using the Applied Biosystems High-Capacity cDNA Reverse Transcription Kit. Gene-specific primers targeting inflammation-related genes (e.g., TNF-α, IL-6, IL-1β, IL-10, and TGF-β) are used with a qPCR mix to amplify the cDNA. Catalog numbers of the TaqMan assays (Thermo Fisher Scientific) used were the following: Tnf Mm00443258 m1, IL1B Mm00434228 m1, IL10Mm01288386 m1, and Actb Mm02619580g1. Following the protocol, the qPCR reaction is performed in a thermal cycler with appropriate controls and replicates. The qPCR (Roche LightCycler 480 II) and cytokine levels (IL-6, TNF-α, and TGF-β) were analyzed using a Biorad system. Quantification is performed using Light Cycler 480 software, release 1.5.0 SP3, version 1.5.039.

### Statistical analysis

All the statistical analyses were done using OriginPro software version 8 and using student’s t-test. All statistical tests were two-tailed, with *p* < 0.05 considered statistically significant. The continuous variables are presented as mean ± standard deviation (SD), while the categorical variables are reported as frequency and mode (percentage). For the study, replicates (*n* = 3), sample sizes (*n* = 6 mice/group), and methods (two-tailed t-test, *p* < 0.05) we used for reproducibility.

## Results

### Physio-chemical characterization

**Fig. 3:**
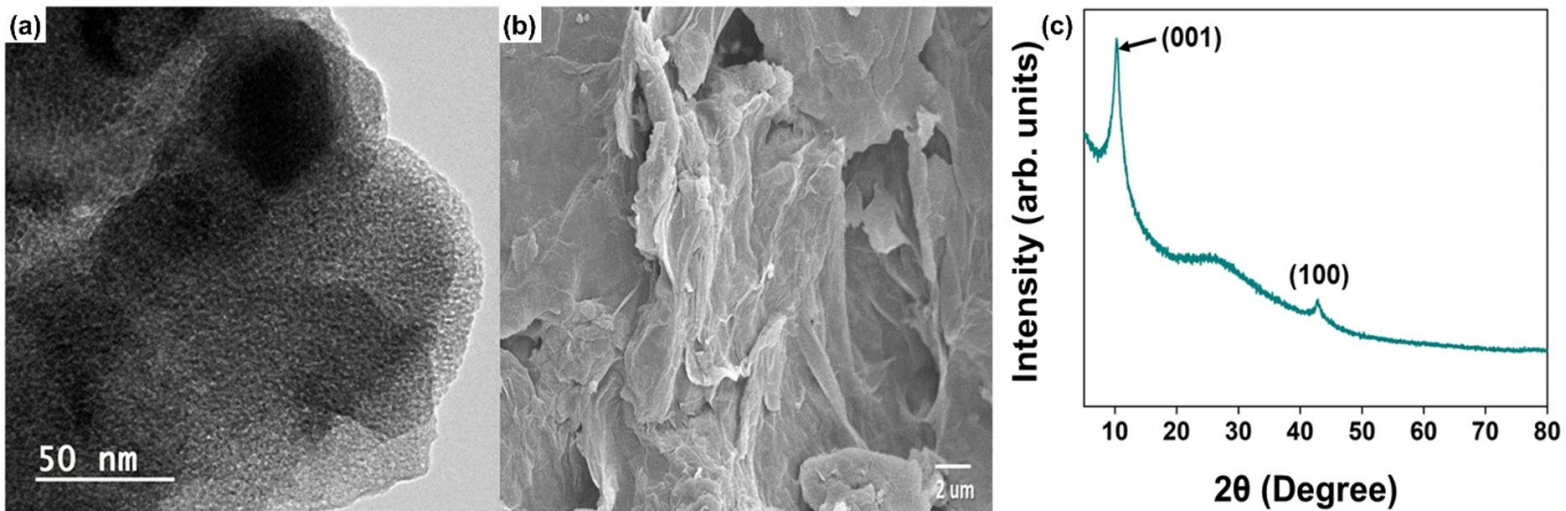
(**a**) TEM images of GO (**b**) SEM images of GO (**c**) X-ray diffraction pattern of graphene oxide (GO).

The structural and morphological characteristics of graphene oxide (GO) were determined using transmission electron microscopy (TEM) and scanning electron microscopy (SEM). A folded flake of a few hundred nanometers in length and breadth can be seen on the lower side of the TEM image (Fig. 3a), suggesting an extremely thin flake. The majority of GO sheets are single or multi-layered. AFM’s morphology and surface topology assessment confirmed that the GO layer thickness is approximately 0.9 nm (Fig. 3). Tiny overlapping sheets observed in TEM analysis are characteristic of graphene oxide (GO) and were absorbed, causing the graphene sheet to distort. A wrinkled surface and a piled-layered structure can be observed in the SEM analysis, which complements the TEM morphology. Figure 3b depicts the puffy nature of GO layers that resulted in agglomerated morphology. The X-ray diffraction (XRD) pattern further substantiated the oxidation process and structural transformation. Pristine graphite typically exhibits a sharp (002) reflection near 2θ ≈ 26°, corresponding to an interlayer spacing of about 0.34 nm. In contrast, the GO sample displayed a broad low-angle peak at around 2θ ≈ 10–12°, indicative of a (001) plane spacing of ~ 0.8 nm (Fig. 3c). The shift and broadening of this reflection suggest an expanded interlayer distance and disrupted π–π stacking due to the insertion of oxygen functionalities and intercalated water molecules.

**Fig. 4:**
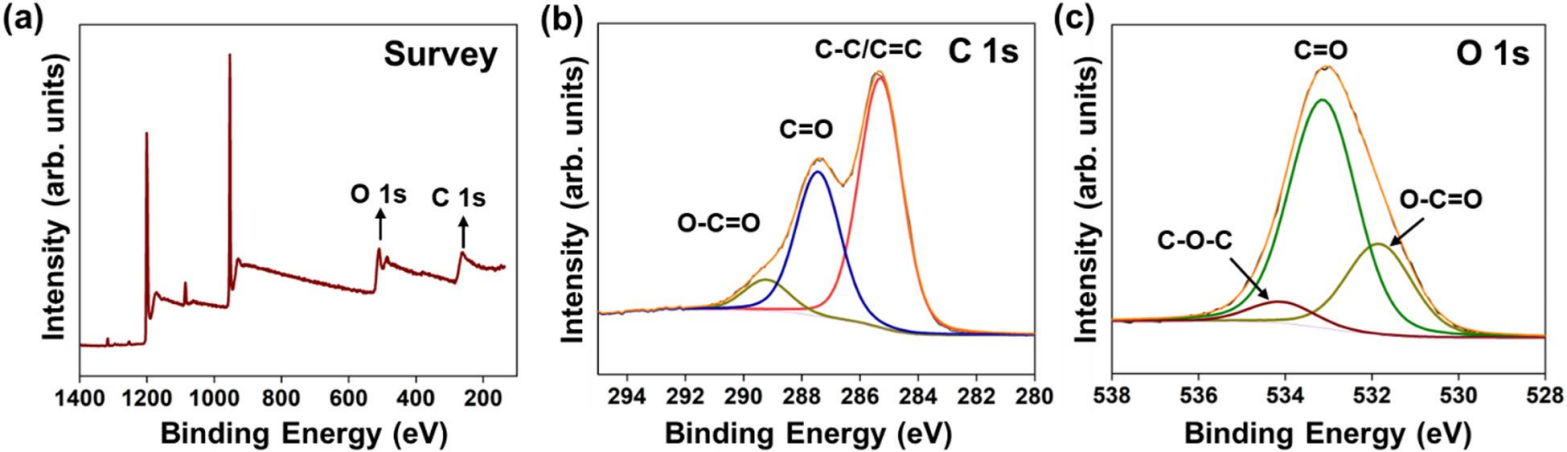
X-ray photoelectron spectrum. (**a**) Survey spectrum showing C and O as the main elements. High resolution (**b**) C 1s and (**c**) O 1s spectra.

The X-ray photoelectron spectroscopy (XPS) survey spectrum of the graphene oxide (GO) sample revealed prominent carbon and oxygen peaks, confirming successful oxidation of graphene sheets. The high-resolution C 1s spectrum was deconvoluted into three components centered at approximately 284.57 eV (sp² C–C/C = C), 287.8 eV (C = O), and 288.7 eV (O–C = O from carboxyl moieties). The relative enrichment of oxygen-containing species indicated substantial functionalization of the graphene lattice with epoxide, hydroxyl, carbonyl, and carboxyl groups. The corresponding O 1s profile exhibited main features near 531–534 eV, consistent with oxygen bound to carbon and adsorbed molecular oxygen or water (i.e., O–C = O, C = O, and C–O–C groups centred at 531.2, 533.1, and 534.1 eV, respectively). The presence of oxygenated carbon species confirms successful oxidation of graphene to GO. These findings collectively verify the introduction of polar oxygen functionalities that increase the hydrophilicity and chemical reactivity of GO (Fig. 4). Together, the XPS and XRD results confirm the successful synthesis of graphene oxide, characterized by extensive surface oxidation and increased interlayer spacing relative to graphite.

### In vitro siRNA release kinetics—effect of drug concentration and pH

**Fig. 5:**
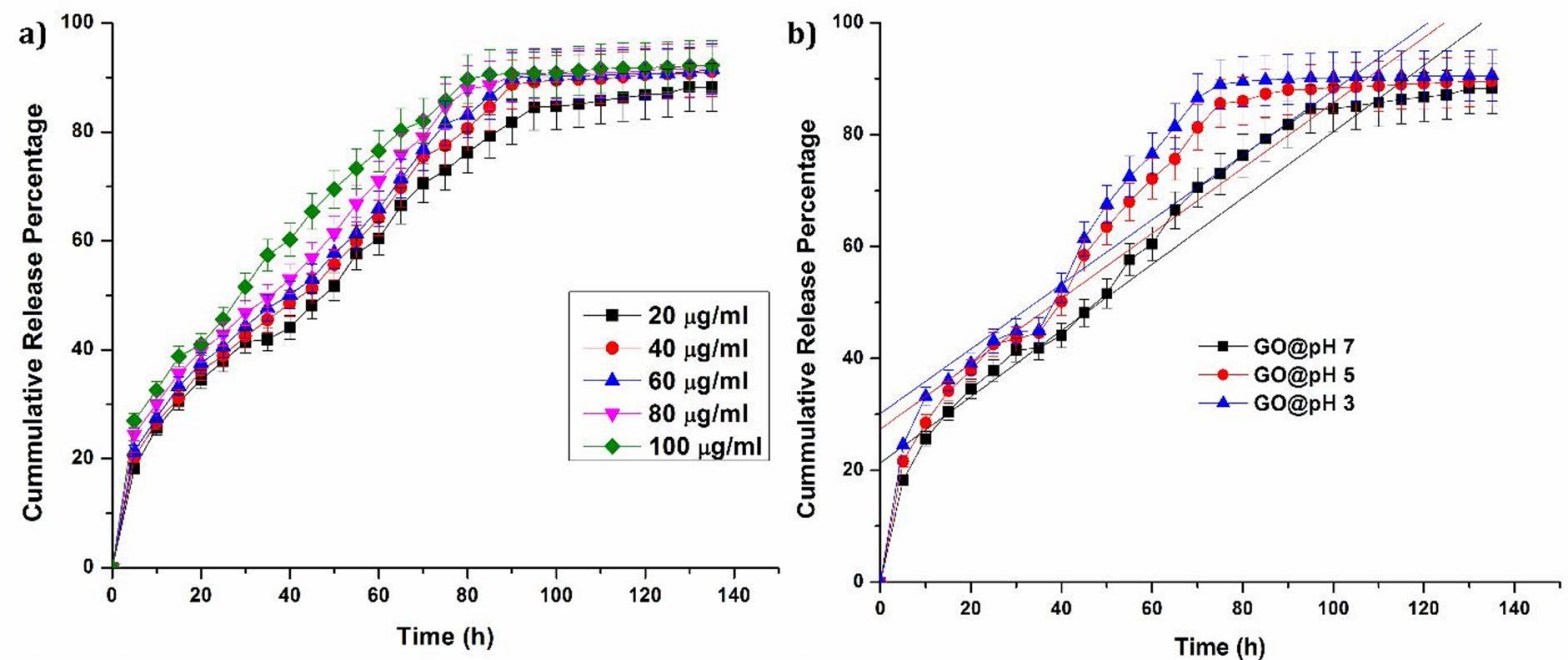
(**a**) The in vitro siRNA release kinetics of GO with varying concentrations of siRNA (**b**) Drug release kinetics of GO with siRNA with varying pHs of 7, 5, and 3 in vitro were fitted into the Peppas model to study the kinetics of the siRNA released (*n* = 3).

The release kinetics of siRNA from GO at neutral pH are illustrated in Fig. 5a, with different siRNA concentrations. The release percentage increased as the concentration of siRNA in GO increased. The time required to achieve 85% siRNA release was reduced when the siRNA concentration was increased, as expected. One of the major observations is the excessive burst release from the GO sheets, which accounts for approximately 35–40% within 30 h (Fig. 5). An important implication of this activity is that it may lead to delivery at non-specific sites. When the GO is exposed to cells, the initial rapid burst release of siRNA may provide a high level of stimulation and trigger a series of cellular reactions; however, persistent siRNA release at a low rate may help sustain stimulation around the site for treatment and osseointegration.

### In vitro bioactivity studies

**Fig. 6:**
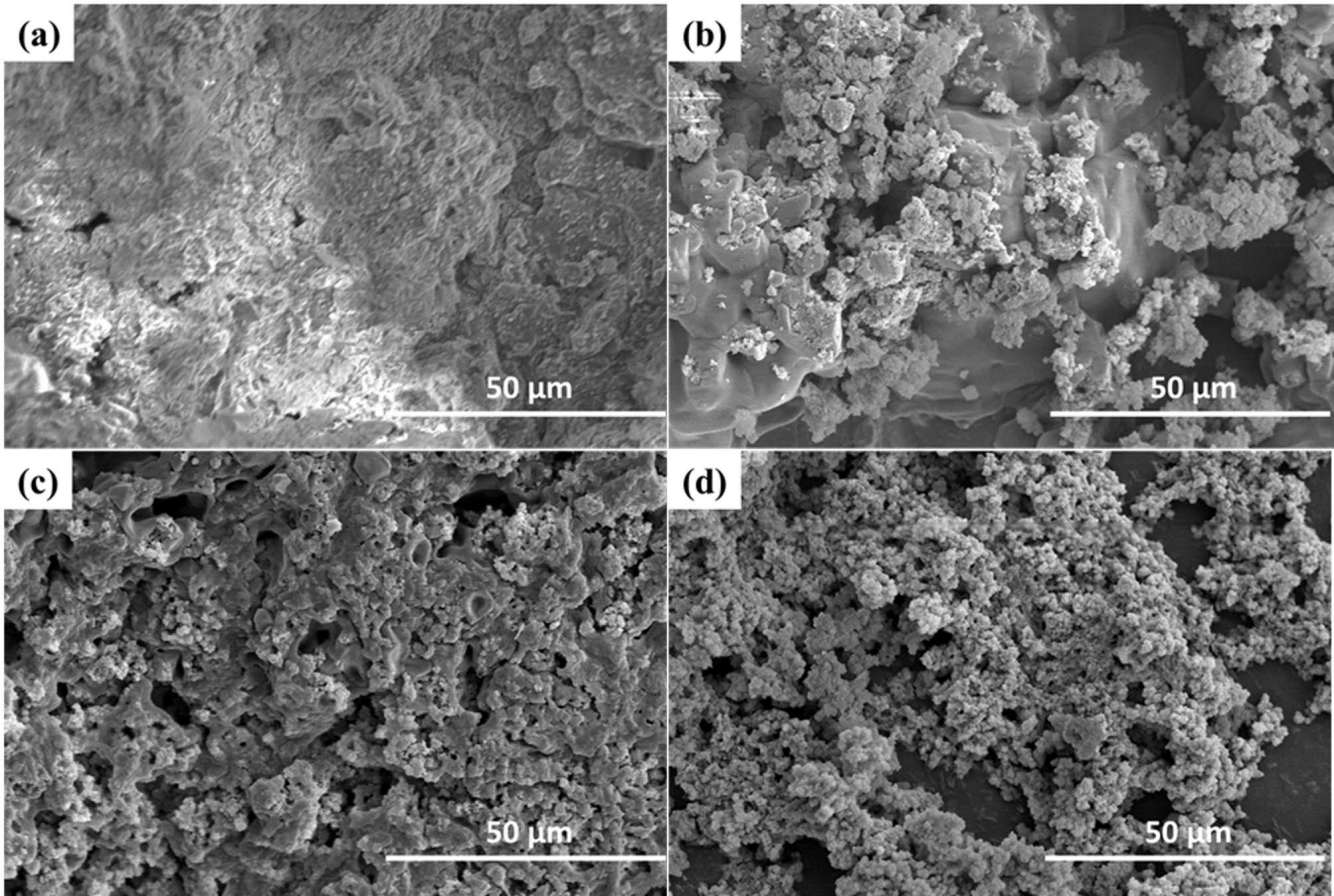
SEM images show the bioactivity of GO after (**a**) 0th -day (**b**) 7th -day (**c**) 14th -day (**d**) 21st -day of immersion in SBF.

**Fig. 7:**
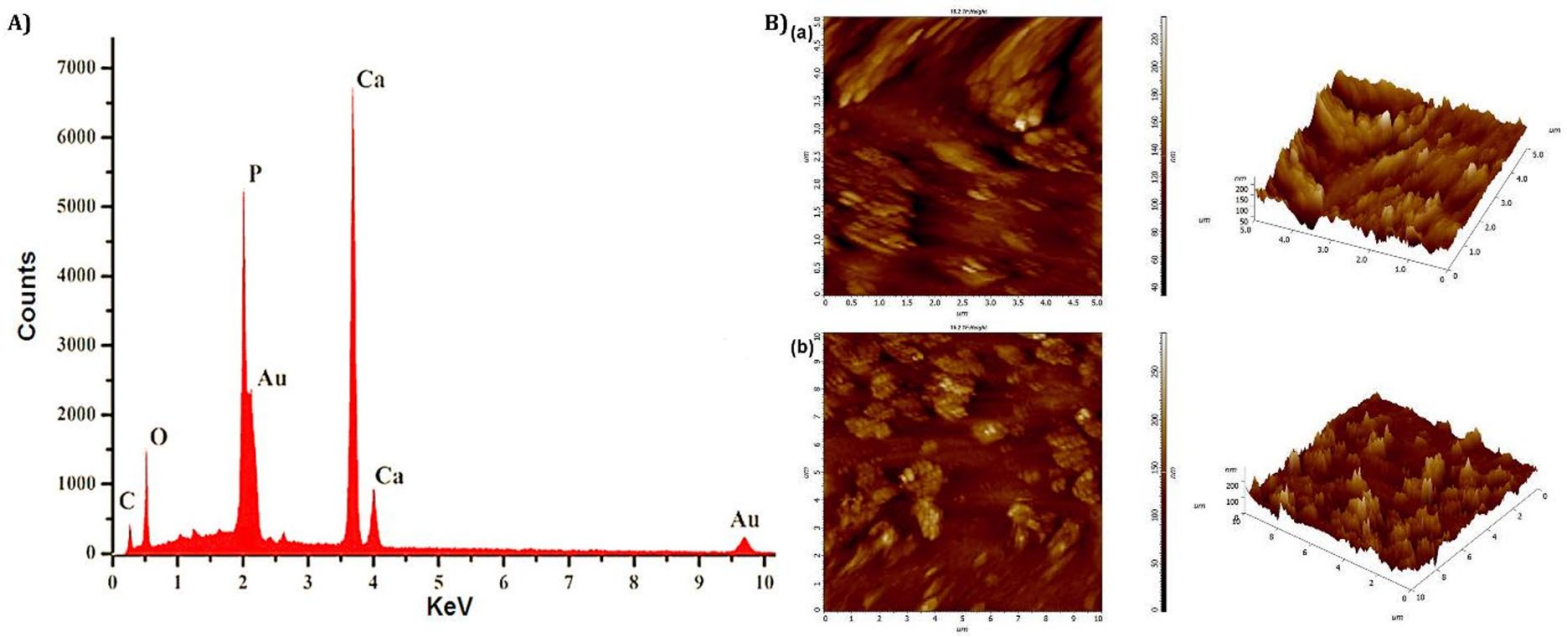
(**A**) EDAX spectra of GO after immersion in SBF for 21 days (**B**) AFM Analysis of GO after 0th, and 21st day of immersion in SBF.

SEM images of GO composite after being immersed in SBF for varying durations are represented in Fig. 6a, b, c, d. The results demonstrate rapid calcium phosphate deposition over the material’s surface during in vitro biomimetic mineralization, indicating potent bioactivity. After the 14th and 21st days in the SBF, a high quantity of sphere-like deposits was discovered, and the size of the deposits was enhanced (Fig. 6c, d). The negatively charged PO_4_^3−^ ions and OH^−^ ions were now attracted to the positively charged GO. The Ca^2+^ ions that were thus adsorbed onto oxygen functional groups served as nucleation sites, directing hydroxyapatite development mostly along the (112) plane. Deposition of calcium phosphate upon the surface of GO/HA composites that had been soaked in SBF for 21 days was detected using EDS. Carbon, oxygen, phosphorus, calcium, and gold elements were observed (Fig. 7A), with the gold element attributable to gold sputtering on the sample during SEM examination. These results have effectively demonstrated that supplementing HA with GO facilitates the generation of bone-like apatite on the surface of GO/HA composites (Fig. 7B). When GO/HA composites were submerged in SBF, GO increased the dissociation of calcium from HA, mainly on the surface of the composites.

### Cytotoxicity activity

#### MTT assay

**Fig. 8:**
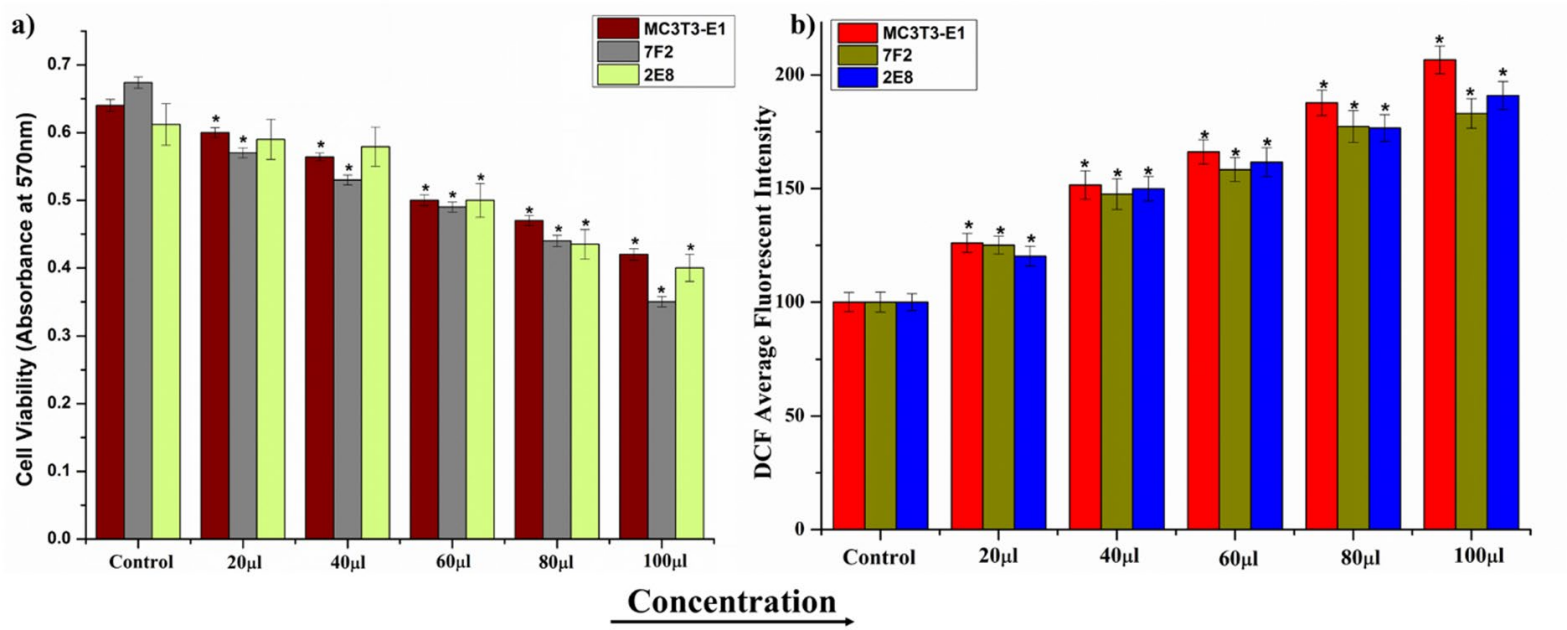
(**a**) MTT assay carried out for cytotoxicity on normal cell lines - in vitro treated GO (**b**) ROS activity on normal cells with different concentrations of GO - in vitro (* denotes the statistically significance of the data, *n* = 3).

**Fig. 9:**
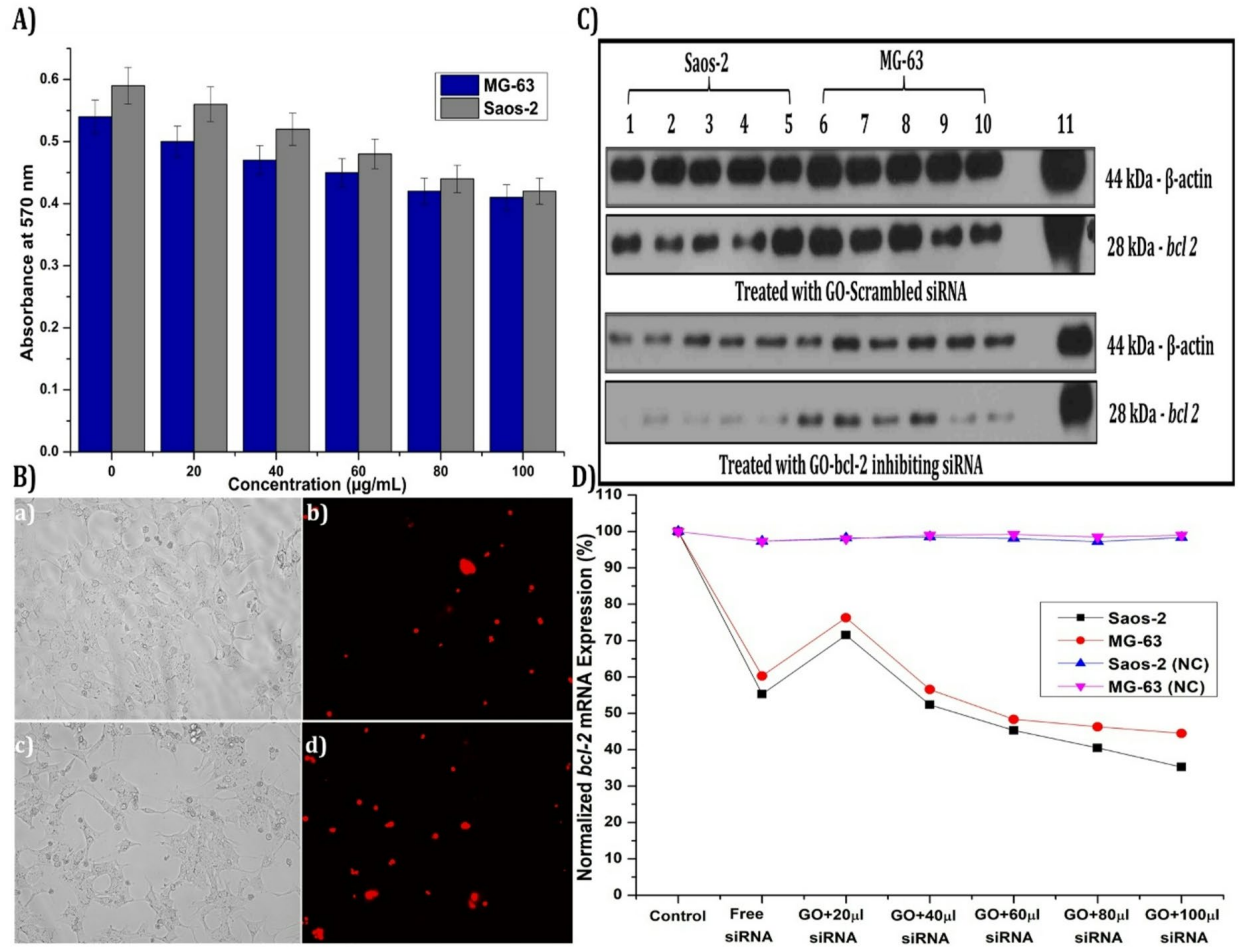
(**A**) Cytotoxic activity using MTT assay on Saos-2 and MG-63 osteosarcoma cells with different concentrations of GO - in vitro; (**B**) Fluorescent microscopic images of MG-63 cells stained with propidium iodide, where (**a**) and (**b**) correspond to the control group and (**c**) and (**d**) correspond to the siRNA-treated cells using GO; (**C**) Western blot analysis of bcl-2 knockdown efficiency mediated by GO-siRNA complexes in Saos-2 (lanes 1–5) and MG-63 (lanes 6–10) cells. β-actin (44 kDa first and third panels) served as loading control; bcl-2 (28 kDa) was probed in both scrambled siRNA-treated groups (second upper panels per cell line) and bcl-2-inhibiting siRNA-treated groups (fourth lower panels). Lanes represent increasing GO-siRNA volumes in the range of 20 µL, 40 µL, 60 µL, 80 µL, and 100 µL (from Lanes 1–5 & Lanes 6–10). Results demonstrate selective, volume-dependent downregulation of bcl-2 protein only. (**D**) Transfection efficacy of GO containing various siRNA concentrations on Saos-2 and MG-63 osteosarcoma cells.

The MTT analysis, which measures cellular mitochondrial activity in a substance, is often used to assess a material’s compatibility effects on cells. For all GO concentrations investigated, the cell viability dropped as the quantity of GO increased, as seen in Fig. 8a. For all MTT concentrations examined, the cumulative proportion of live cells on control has always been greater than on GO. In MCF-7 cells, Gurunathan et al.^34^ observed that GO can reduce cell viability depending on dose. A dosage of > 60 g/mL of GO causes substantial cytotoxicity. This dose-dependent profile underscores GO’s relative inertness at lower, therapeutic levels (20–100 µM siRNA equivalents, where viability remains > 80%), as evidenced by minimal impact in osteosarcoma-targeted assays (Fig. 9a), while higher doses elevate ROS (Fig. 8b) but are non-relevant for clinical translation. In this study, GO exhibited high biocompatibility, with significant cytotoxic effects appearing only at higher concentrations. The use of WST-1 was noted as an alternative viability assay recommended for two-dimensional (2D) nanomaterials due to their potential interference with MTT reagents^34^.

#### ROS assay

ROS assay results are shown in Fig. 6b. The oxidative stress on the cells increased exponentially with a rise in the concentration of GO. The results agreed with the cytotoxicity results (Fig. 9a). This may be the reason for the reduction in cell viability in the cytotoxicity assay. ROS level was slightly higher compared with the control (Fig. 9b). However, this ROS elevation is primarily at supra-therapeutic GO concentrations (> 60 µg/mL) and is attenuated in siRNA-complexed formulations, where targeted bcl-2 modulation shifts cytotoxicity toward cancer cells, preserving normal cell viability.

### Cytotoxic activity

The GO loaded with siRNA for cytotoxic activity on osteosarcoma cells (Saos-2 and MG-63) treated with various dosages of GO for 12 h was analyzed using the MTT assay. The results demonstrated that cell viability was reduced at all doses of GO-siRNA, varying from 20 to 100 µL of siRNA (Fig. 9A). These in vitro findings suggest targeted cytotoxicity via bcl-2 silencing but require corroboration in tumour-bearing models to assess therapeutic relevance. From the blots, it was observed that there was a clear decrease in the intensity of the bcl-2 expression in both Saos-2 and MG-63 cells when treated with GO loaded with bcl-2 inhibiting siRNA, and this drop became more obvious starting with the increase in concentration. Whereas the β-actin bands, on the other hand, held steady in strength no matter the treatment or dose, which tells us the loading was even and the changes weren’t from some nonspecific cell damage. In the parallel lanes where GO carried scrambled siRNA, the bcl-2 bands looked almost unchanged right up to the highest concentration, showing the effect really came from the specific bcl-2 sequence causing the inhibition of bcl-2. Comparing the two cell types, Saos-2 seemed to lose a bit more bcl-2 protein than MG-63, but both showed that same pattern of gradual band weakening tied to increasing siRNA amounts (Fig. 9C).

### In vivo Inflammatory responses

#### FACS analysis

**Fig. 10:**
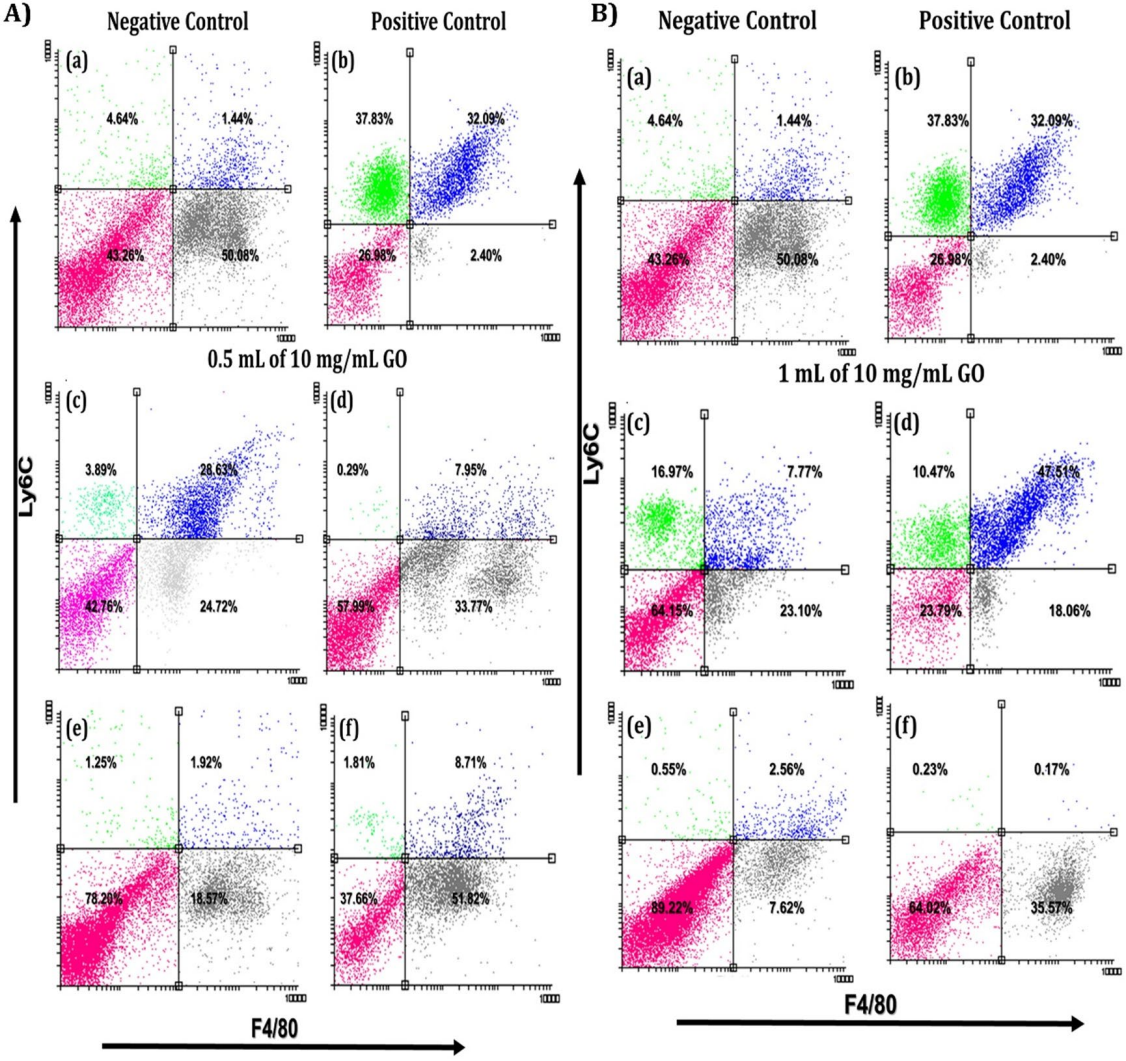
(**A**, **B**) FACS analysis of samples isolated from the peritoneal fluid collected from the mice sacrificed after treating with 0.5mL of 10 mg/mL of GO & 1mL of 10 mg/mL of GO, Panels: (**a**) Untreated/negative control (healthy, un injected mice; baseline ~ 5% Ly6C^high^); (**b**) Positive control (LPS 20 µg/mL; sustained ~ 38% Ly6C^high^); (**c**–**f**) GO-treated (0.5/1 mL, 10 mg/mL) at 7, 14, 21, 28 days. Gating: Pink – Ly6C^low^ & F4/80^low^ (monocytes/neutrophils); Grey – F4/80^high^ Ly6C^low^ (resident macrophages); Blue – Ly6C^high^ F4/80^high^ (inflammatory macrophages); Green – Ly6C^high^ F4/80^low^ (neutrophils). *n* = 6/group.

The in vivo inflammatory response of GO was assessed by injecting 0.5 mL and 1 mL of 10 mg/mL GO into the peritoneal cavity of mice (Fig. 10A, B), followed by observation at various time intervals, as described in the materials section. This non-tumour model prioritizes biocompatibility evaluation over disease-specific efficacy, complementing in vitro cytotoxic insights while highlighting the need for orthotopic osteosarcoma contexts in future work. Ly6C and F4/80 stains were used to assess inflammation in peritoneal fluid collected from mice with and without GO dosing for a defined period as mentioned in methods. Compared to untreated (healthy) and placebo (PBS) controls—which showed baseline myeloid profiles (e.g., ~ 5% Ly6C^high^ neutrophils)—GO treatment induced transient elevations (peaking 7–14 days, e.g., 6–18% neutrophils at 0.5-1 mL doses), resolving by 28 days and distinct from LPS-positive control’s sustained inflammation (~ 38% neutrophils) (Fig. 10A (b), B (b)). This highlights GO-specific, adaptive effects without chronic activation. FACS was able to extract each myeloid cell type using this panel and methodology. Although F4/80 expression has been regarded as a marker for mouse tissue macrophage populations that arise through embryonic cells that regenerate independently of blood monocytes, our study was focused only on the macrophage population. Based on the level of macrophage expression, these peritoneal macrophages are classified into big peritoneal macrophages expressing high amounts of F4/80 and tiny peritoneal macrophages expressing low levels of F4/80. Ly6C is expressed on neutrophils, monocytes, dendritic cells, and subsets of CD4 + and CD8 + T cells, which we utilized to identify the presence of neutrophils in the current study (Fig. 10A, B).

#### Gene expression

Cells were collected from the peritoneal fluid of the GO-treated and untreated mice over a period. Using qPCR, the amounts of *IL-6*, *TNF-α*, *TGF-β*, *IL-1α*, *IL-10*, and *MIP-1β* were determined. Cytokines are immunomodulatory proteins that aid in coordinating the immune response to an extensive range of inflammatory activities, including infection. In mouse models, cytokines such as *IL-6*, *TNF-α*, and *TGF-β* have been shown to be integral to the host immunological response to intracellular infections^35–37^. Inflammation can potentially alter the expression of cytokine receptors on target cells. Hence, we choose to evaluate the mice’s expression of TNF-α and IL-6 receptors.

**Fig. 11:**
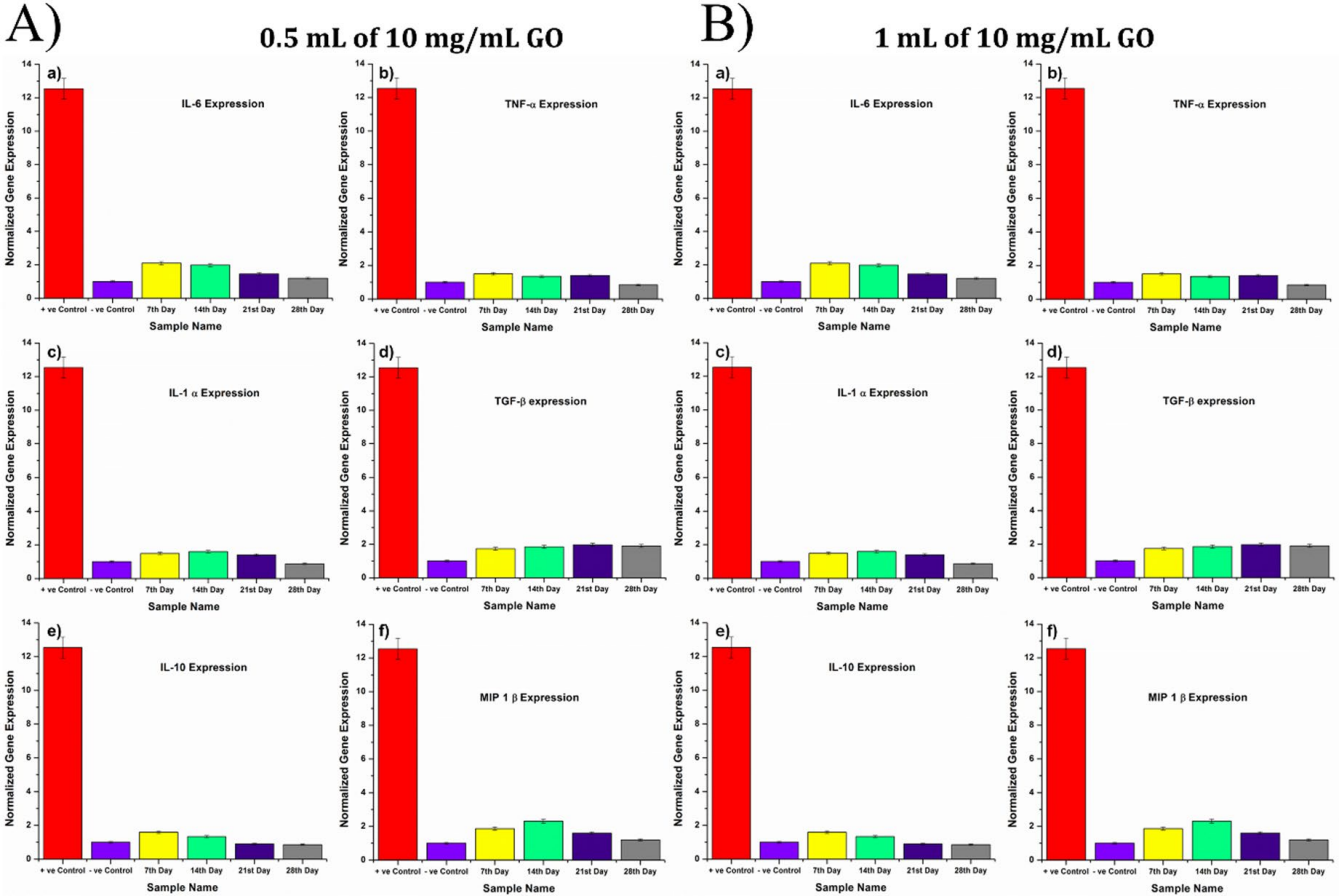
The gene expression of the inflammatory cytokines tested on the samples isolated from the peritoneal fluid collected from the mice sacrificed at different periods after treating with (**A**) 0.5mL of 10 mg/mL of GO in mice (**B**) 1 mL of 10 mg/mL of GO in mice.

When incubated with 0.5 mL of 10 mg/mL GO, the level of inflammatory cytokines expressed was minimal compared to untreated/healthy and PBS placebo controls (fold-change ~ 1.0 across timepoints), while LPS-positive controls exhibited persistent upregulation (e.g., IL-6 > 5-fold at 28 days). GO dosing yielded early peaks (e.g., 3-4-fold for IL-6/TNF-α at 7 days) that declined to baseline by 28 days (Fig. 11), underscoring transient, GO-specific immunomodulation. After the 7th day of incubation with GO, the expression of IL-1α, IL-10, and MIP-1β nearly quadrupled and continued until the 28th day. The early upregulation of cytokines within 7–14 days, followed by a downregulation of the same, suggests that inflammation may have occurred at the initial stage of exposure to GO (Fig. 11A & B).

This was evident from the FACS, where neutrophils and monocytes were initially observed between 7 and 14 days of incubation of mice with GO at both concentrations. The same is observed in the regulation of the cytokine, which shows that both results are comparable. Feito et al.^38^ reported that GO functionalization with biocompatible polymers is more stable under physiological conditions and reduces immune reactions by limiting interactions with other biomolecules^38^.

## Discussion

The study on evaluating the inflammatory responses of GO is a novel approach in biomedical research due to its importance. The ainano-sized channels between the graphene oxide (GO) layers enable small molecules, such as water molecules, to pass through them. Bai et al.^39^ synthesized graphene oxide (GO) using Hummers’ method, and their findings indicate that the GO exhibited somewhat folded morphologies with wrinkles similar to those observed in the GO synthesized in our study^39^. The release was rapid when the GO was loaded with siRNA at different concentrations. This may be because siRNA may have become more hydrophilic and water-soluble at low pH, resulting in increased release. Furthermore, because siRNA contains an NH_2_ group, its hydrophilicity increases, which explains why the release ratio is higher in acidic conditions. In a therapeutic setting, the pH-dependent release of medication from GO is crucial because the microenvironments of tumour extracellular tissues, intracellular lysosomes, and endosomes are acidic^40^. The in vitro siRNA release kinetics in GO with different pH values showed that the release of siRNA was approximately 35–40% at various pH levels (Fig. 5b). The initial release was attributed to the burst phase, which constituted approximately 40–45% of the overall siRNA concentration (Fig. 5b). The burst release occurred due to the siRNA on the surface of the graphene oxide (GO) sheets. Reports suggest that the burst release mitigated via LbL polymer coatings^26,40^, reducing non-specific delivery while preserving ~ 85% sustained release. It is to be noted that GO colloids exhibit greater stability within the pH range of 3 to 11, as reported by numerous researchers. The thickness of the GO sheets is typically expected to increase as the pH decreases due to heightened protonation of the acidic groups (C–OH, COOH) present on the sheets. This might have led to the entrapment of siRNA inside the GO sheets as pH increased. Similarly, due to the accumulated negative charge on GO sheets and repulsive interactions between them, the colloidal stability of GO in an aqueous medium is electrostatic. The -COOH groups on the GO surface tend to be protonated, meaning they acquire H+ from the medium when the pH decreases. Protonation is reported to reduce the overall charge on the sheets, decreasing the electrostatic repulsion between them. This eventually leads to sheet aggregation and a reduction in colloidal stability^41^. The release of siRNA from GO and the effect of pH may be due to the above-discussed reasons. Furthermore, the defects of exposed GO in interaction with SBF function like nucleation sites, as indicated by the ability of the as-expected GO to form apatite on its surfaces. Because of the increased calcium ion concentration in SBF, the greater negative charge, and more accessible nucleation sites, GO/HA composites may rapidly generate bone-like apatite on the sample’s surface, mimicking natural bone. AFM studies further confirmed the formation of apatite on the GO, as the surface roughness increased by approximately ± 100 nm after soaking in SBF for 21 days, whereas this was not the case on the 0th day (Fig. 7B). Thus, all these data demonstrate that the material is bioactive and will facilitate osseointegration when applied in vivo, provided suitable biocompatibility issues are addressed. Furthermore, the relative growth rates of the GO groups were lower compared to those of the CS NPs observed in our previous study^32^, with the greatest being approximately 1.5 times that observed in GO cell viability. As a result, functionalizing graphene oxide (GO) with a biopolymer can enhance its biocompatibility. Nevertheless, considering graphene’s biological properties, several studies have demonstrated that nanomaterials can be harmful^42,43^. One of the primary reasons for cytotoxicity may be the interaction of graphene-based compounds with the cell membrane^44,45^. Interestingly, despite GO’s hydrophilic nature, smooth edges, and high oxygen content—attributes that confer generally low toxicity at therapeutic doses—we observed dose-dependent cytotoxicity in normal cells, significant only at concentrations > 60 µg/mL (Fig. 8a), which is mitigated by siRNA synergy in cancer-targeted applications. This observation reconciles with GO’s established inertness by highlighting its dose- and context-dependency: at therapeutic siRNA doses (20–100 µM, Fig. 8a), cytotoxicity is minimal (< 20% viability loss), aligning with biocompatibility benchmarks^34,44^. Significant effects emerge only at higher concentrations (> 60 µg/mL), driven by ROS accumulation (Fig. 8b), but are mitigated in GO-siRNA complexes via specific bcl-2 targeting, which synergistically enhances selectivity for osteosarcoma cells (e.g., ~ 65% knockdown with retained normal viability, Fig. 9A, C). Thus, while raw GO exhibits concentration-specific toxicity, its formulation as an siRNA carrier leverages these properties for controlled, low-risk delivery, warranting surface modifications (e.g., PEGylation^40^ for further optimization. Naked siRNA controls were omitted due to known inefficiency and validated by comparative efficiency^40^. It was observed that GO had higher toxicity than CS NPs compared to our previous studies. However, according to Dudek et al.^46^, GO’s strong affinity for cell membranes may cause cell death by interfering with cell metabolism^46^. The excessive generation of reactive oxygen species (ROS) has been shown to impact the stability of DNA and RNA. Furthermore, mitochondrial dysfunction is associated with the production of reactive oxygen species (ROS) and alterations in mitochondrial membrane potential, both of which can lead to cell death^43^. Earlier research has shown that GO toxicity is linked to lipid peroxidation and oxidative stress caused by the production of reactive oxygen species. For example, Zhang et al.^47^ reported that GO treatment substantially raises ROS levels^47^, while Chatterjee et al.^48^ found that GO can induce oxidative stress in HepG2 cells by enhancing ROS generation^48^. The increased capability of macrophages to absorb GO might have influenced the assembly of actin within the cell, resulting in a reduction in cell survival, as reported by Feito et al.^38^. GO can be easily cross-linked with various materials, including quantum dots, polymers, DNA, biomolecules, and proteins. This reduces aggregation in saline and other biological fluids, enhancing biocompatibility and addressing these limitations^49^. These results indicated that siRNA-loaded graphene oxide (GO) was effective in inhibiting tumour growth. Since these studies have been carried out in vitro, the viability of cells was reduced to some extent; however, this will not be the case when the load is delivered in vivo, due to various defense mechanisms of our immune system^50^. The culture medium contained free siRNA, which resulted in a nearly 50% reduction in mRNA expression in Saos-2 cells, and a similar significant difference was observed in the case of MG-63 cells, at 65%. The viability of *MG-63* cells was not at an appreciable level (Fig. 9A). The results for cytotoxic activity in vitro were supported by the transfection efficacy results as well. When transfection was done with GO containing 20µL of siRNA, *Bcl-2* mRNA expression was higher, as seen in Fig. 9D. As with all concentrations, this was observed to be decreased, as the siRNA concentration was increased (Fig. 9D), but the effectiveness of transfection was completely less. Regardless of the two osteosarcoma cells, mRNA expression of Bcl-2 was inversely related to the siRNA concentration to a certain extent. When comparing Saos-2 cells to MG-63 cells, the transfection efficiency was the same in both cell lines. This further confirms that GO contributes toxicity, but siRNA enhances via specific knockdown (Fig. 9D), synergizing for targeted death without invalidating carrier role. From the blotting analysis (Fig. 9C) it was observed that the scrambled siRNA a nice, sharp Bcl-2 band at the expected ~ 26 kDa in both cell lines (lanes 1–10) intensity looks pretty much like control. Whereas in GO with the bcl-2 inhibiting siRNA treatments the bands get noticeably smeared, weaker, or pretty much disappear in several lanes. It’s not a clean total wipeout in every replicate, but the consistent fading/absence compared to scrambled clearly shows the specific siRNA is effectively inhibiting the bcl-2 expression in par with the MTT assay (Fig. 9C and D). This protein-level loss lines up well with the mRNA drop we already had in Fig. 9D (down to 35–40% at best doses) gives the evidence that the observed viability is tied to actual bcl-2 suppression. Further studies are still needed to know and confirm the exact death mechanism (apoptosis vs. other paths). Additionally, in PI staining of MG-63 cells (Fig. 9B), the viability of the cells was high, confirming that GO, on its own, is ineffective in targeting individual cells in vitro. Myeloid cells inside the bone marrow appear significantly as they differentiate into neutrophils and monocytes. Because of its response components in the promoter region, these Ly6C expressions can be triggered by type I and type II interferons in addition to the intrinsic expression profile^51,52^. Neutrophils conversely express Ly6C uniformly throughout the bone marrow and circulation, and the quantity of Ly6C expression is utilized to differentiate monocyte subsets. Ly6C monocytes (also referred to as immature or inflammatory monocytes) migrate from the bone marrow to sites of infection or inflammation^53,54^. Monocytes and macrophages can be classified as inflammatory (Ly6C^high^), non-inflammatory (Ly6C^low/neg^), or resident (Ly6C^low/neg^) based on the expression of the Ly6C antigen^55^. The differential expression of specific markers distinguishes resident macrophages in this example, characterized as F4/80high and Ly6C low. With increasing incubation duration, the frequency of F4/80^low^ Ly6C^high^ related to monocytes and neutrophils progressively increased. High Ly6C levels (F4/80^low^ Ly6C^high^) further differentiated the circulating neutrophils and monocytes. Positive control (Fig. 10A(b) & B(b)) revealed 32.09% of cells with Ly6C^high^ & F4/80^high^ expression owing to monocytes and 37.83% with Ly6C^high^ and F4/80^low^ expression due to neutrophils. In naive animals, peritoneal macrophages are frequently classified as tissue-resident cells with an F4/80^high^ phenotype, as shown in Fig. 10A(a) & B(a). In contrast, those from thioglycolate-elicited mice (Fig. 10A(b) & B(b)) have a monocyte-derived Ly6C^high^ and F4/80^high^ phenotype and are referred to as inflammatory macrophages. Similarly, the control group had a lower percentage of neutrophils than the positive control, and the positive control had a lower percentage of peritoneal macrophages. Ghosn et al.(2010) and Cassado et al. (2015) found that morphological investigations categorized peritoneal macrophages with F4/80 high and F4/80 low expression as LPM and SPM, respectively^56^. After the 7th, 14th, 21^st,^ and 28th days, mice treated with 0.5 mL of 5 mg/mL of GO had 6.40, 1.23, 1.25, and 3.08% Ly6C^high^ neutrophils, respectively (Fig. 10A (c, d, e, and f)). Similarly, when injected with 1 mL of 10 mg/mL of GO, the number of neutrophils observed from the peritoneal cells sacrificed after the 7th, 14th, 21^st,^ and 28th days showed 7.73, 17.90, 0.55, and 0.23% Ly6C^high^ (Fig. 10B (c, d, e, and f)) expression, which is negligible compared to the positive control. It was observed that the number of neutrophils was high during the initial phase of the study. Still, as the days progressed, it got subdued, which is a concrete fact to be considered for drug delivery and implant application. When GO was injected in vivo in the intraperitoneal cavity, resident macrophages, that is, F4/80^high^Ly6C^low^ under homeostatic conditions, were visible in most of the cases compared to the positive controls (Fig. 10A(b) & B(b)), which further confirms that GO caused slight or no inflammation when compared to the positive control. Another interesting finding was that as the quantity of GO increased, the percentage of neutrophils (F4/80low Ly6Chigh) dropped, which was lower than in the control groups (Fig. 10A & B–(a & b)). This was the same in both 0.5 and 1 mL of 10 mg/mL of GO after injecting mice. However, regardless of the concentration of GO, the fraction of cells exhibiting inflammatory monocytes (F4/80^low^ Ly6C^high^) was reduced. On the 7th day of exposure to 0.5 mL of 10 mg/mL GO, a maximum of 20.32% (Ly6Chigh, F4/80high) was detected. However, after 14 days of incubation with 1 mL of 10 mg/mL GO, the percentage increased to 30.64%. It was evident from the protein expression that as the concentration of GO increased, the number of inflammatory monocytes (F4/80^high^ Ly6C^high^) and neutrophils (F4/80^low^ Ly6C^high^) decreased exponentially with the increase in exposure duration. Furthermore, compared to the positive, the percentage of F4/80^high^ Ly6C^high^ was reduced, an important factor for in vivo applications. Considering the results from the protein expression, it is clear that the use of GO has not caused any inflammation on exposure to the mice. In the present case, the upregulation of inflammatory cytokines in the initial stage was possible because GO might have been identified by the complement system components as soon as it was introduced into a biological system. MIP-1β is a chemokine produced by various types of cells, including monocytes, activated T cells, and B cells. Consequently, the MIP-1β and other gene expressions are similar to our FACS results, as the amount of expression upregulated during the initial phase of incubation with GO is similar to the greater level of monocytes and neutrophils during the 7th and 14th days in FACS. The increase in all these cytokines levels compared to the control was attributed to a compensatory or adaptive immune response. However, the downregulation of expression after the 14th day may be related to immune system decompensation or failure that occurs after or following extreme stress. This pattern of regulatory changes in both concentrations over time indicates the immune system’s attempt to reestablish homeostasis, but in this case, it occurs more rapidly. *IL-10* is a cytokine recognized for its potent anti-inflammatory properties, the production of inflammatory cytokines by activated macrophages, including *TNF-α*, *IL-6*, and *IL-1α*. However, unlike many biopolymers, GO at higher concentrations exhibited a downregulated level of IL-10, significantly increasing the normalized gene expression of IL-1α, IL-6, and TNF-α over 28 days, while *IL-10* expression increased. Although the level of these cytokine expressions was lower in all cases, the results were comparable to those of other researchers^57–59^. Even at lower concentrations, the level of *IL-10* after the 21st day was consistent with the downregulation of other cytokine levels. *TNF-α* is a well-known cytokine that has both beneficial and potentially lethal pro-inflammatory and cytotoxic effects in immune regulation and immunological responses. These effects are downregulated over a longer duration of exposure with GO. *TGF-β*, on the other hand, reduces cytokine production by inhibiting macrophage and Th1 cell activity; suppresses the production of IL-1α, IL-2, IL-6, and TNF-α; and activates an *IL-1α* receptor antagonist, which blocks *IL-1α* expression. From the gene expression studies, it was clear that after 7–14 days of exposure, the pattern of cytokines declined, with an increase in TGF-β, and the levels of other cytokines were negligible at both concentrations after GO injection. Among these, *IL-6* plays a significant role, as it predominantly promotes pro-inflammatory signalling and influences numerous cellular processes. The qPCR studies for the cytokine expression obtained at different times agreed with the FACS analysis (Fig. 11). Moreover, GO–siRNA complexes were formed via π–π stacking interactions^40^ hence subsequent immune interaction occurred through complement recognition, leading to transient Ly6C upregulation^46,50^, which later resolved through TGF-β–mediated suppression (Fig. 11). Thus, this confirms the ability of GO to sustain itself within the bodies of mice without causing any major inflammation, which is a positive sign for its use in further applications. While GO-alone serves as a practical proxy for non-targeting controls—evidenced by unaltered bcl-2 mRNA (Fig. 9D) and high PI viability (Fig. 9B) indicating non-specific effects are minimal—the absence of GO-loaded scrambled siRNA precludes definitive distinction between bcl-2-specific silencing and potential complex-induced cytotoxicity. This aligns with guidelines recommending scrambled sequences as essential negative controls in cancer RNAi experiments to validate on-target mechanisms. Therapeutic claims are thus framed as preliminary insights into modulation efficacy, warranting future studies incorporating such controls alongside advanced nanocarrier optimizations for siRNA delivery in osteosarcoma.

Exciting new developments in using exosomes to deliver siRNA are really boosting the promise of nanocarrier approaches for tweaking genes—much like the graphene oxide (GO) setup we explored in this study. Qiu’s et al.^60^ found that exosomes from milk, loaded with siTGF-β1 and delivered via nebulizer, helped ease lung fibrosis. It worked by shutting down the EMT pathway that promotes scarring and making it easier for collagen to break down. Recently, Yuan et al. (2025a, 2025b) demonstrated that functionalized exosomes loaded with siFKBP10 or siHSP47 effectively inhibited collagen biosynthesis and fibroblast differentiation, respectively, by silencing profibrotic genes, thereby illustrating the therapeutic potential of targeted siRNA delivery. Finally, it can be concluded that blending GO platforms with natural vesicles could enhance siRNA encapsulation thereby they can serve accurate, and deliver better results against complex diseases like osteosarcoma^60–62^. One of the key limitations of our study lies in the methodological disconnect between its in vitro and in vivo components. While in vitro experiments effectively demonstrate GO-siRNA’s potential for bcl-2 regulation and cytotoxicity (using MTT assay) in osteosarcoma cell lines (Fig. 9A, D). Meanwhile, the in vivo work relied on a tumour-free intraperitoneal injection setup to assess body-wide inflammation and overall compatibility (Figs. 10 and 11). Importantly, our study elucidates GO’s general immune dynamics—such as transient neutrophil modulation and cytokine resolution—supporting its safety profile as a carrier. However, it does not replicate the osteosarcoma tumour microenvironment, limiting direct testing of the therapeutic hypothesis (e.g., anti-tumour efficacy). It was observed that the burst release of 35–40% is attributed to the surface modification on GO to attain a more sustained release in tumour-site-specific release in acidic environments. Additional investigations have to be incorporated with osteosarcoma models in vivo to evaluate bcl-2 knockdown, anti-cancer effects, and osseointegration observed in vitro from GO’s bioactivity. Consequently, claims of osteosarcoma treatment potential are preliminary and further studies using an orthotopic or xenograft tumour-bearing models would be needed for translational validation, to understand the tumour progression, metastasis, and targeted delivery [e.g.,^12,16^]. Future studies integrating these models could bridge this gap, enhancing GO-siRNA’s applicability.

## Conclusion

The results from this work demonstrate that GO holds great potential as a carrier for siRNA delivery, exhibiting promising efficiency both in *vitro* and in vivo. During the in vitro siRNA release, the major drawback observed was that excessive burst release from the GO sheets accounted for approximately 35–40% within 30 h. This is an essential factor that needs to be considered since it may lead to delivery at non-specific sites. GO has demonstrated its ability to protect the drug from lysosomal attack, which aligns with the findings of other researchers. Interestingly, despite GO’s hydrophilic nature, smooth edges, and high oxygen content—attributes that confer generally low toxicity at therapeutic doses—we observed dose-dependent cytotoxicity in normal cells, significant only at concentrations > 60 µg/mL, which is mitigated by siRNA synergy in cancer-targeted applications. The results of the MTT assay showed lower viability of normal cells with GO compared to many biocompatible polymers, which is negligible and can be overcome through functionalization with biopolymers like chitosan, which in turn also reduces the burst release of the drug significantly. In PI staining of MG-63 cells, viability was comparatively higher, confirming GO’s relative inertness in isolation at therapeutic doses, though siRNA complexation enhances targeted efficacy without disproportionate normal cell damage. The biocompatibility and large surface area of GO enable the efficient loading of siRNA molecules and successful delivery to target cells, thereby increasing the efficacy of gene silencing. Nevertheless, the inflammation discussed, especially in the case of in vivo studies, underscores the need for further cautious approaches to dosage and surface changes to prevent excessive immune responses. More extensive investigations are needed while designing the carrier, considering immunotoxicity issues associated with the GO-siRNA delivery system. This presents a promising opportunity for utilizing the GO-based siRNA delivery system in clinical treatment. Still, further studies are needed before applying it to various applications, such as controlling the drug’s burst release and increasing the GO concentration, which could lead to a potential carrier that provides a synergistic effect with biocompatibility and bioactivity.

## Supplementary Information

Below is the link to the electronic supplementary material.

Supplementary Material 1

## Data Availability

All the data for the study is provided within the manuscript.
