# Supplementary material for: In vitro bioactivity, cytotoxicity, and gene silencing of graphene oxide as a Bcl-2 siRNA carrier in osteosarcoma cells with in vivo inflammatory response
Source: Sci Rep. 2026 Apr 17;16:17810. doi: 10.1038/s41598-026-48934-0 (PMC13247253; doi:10.1038/s41598-026-48934-0)
Supplement: Supplementary file 1 — Supplementary Material 1 [file 41598_2026_48934_MOESM1_ESM.pdf]

SUPPLEMENTARY DATA

Table S1: Quantitative Data for MTT Assay for different cell lines used with p-Value

Assay	Cell Line	siRNA Concentration (μM)	Viability/Expression (% or Fold Change)	p-value (vs. Control)
MTT (Cytotoxicity, GO alone)	MC3T3-E1	0 (Control)	100%	-
MTT (Cytotoxicity, GO alone)	MC3T3-E1	20	95%	p > 0.05
MTT (Cytotoxicity, GO alone)	MC3T3-E1	40	85%	p < 0.05
MTT (Cytotoxicity, GO alone)	MC3T3-E1	60	70%	p < 0.01
MTT (Cytotoxicity, GO alone)	MC3T3-E1	80	60%	p < 0.001
MTT (Cytotoxicity, GO alone)	MC3T3-E1	100	50%	p < 0.001
MTT (Anticancer, GO-siRNA)	Saos-2	0 (Control)	100%	-
MTT (Anticancer, GO-siRNA)	Saos-2	20	90%	p < 0.05
MTT (Anticancer, GO-siRNA)	Saos-2	40	80%	p < 0.01
MTT (Anticancer, GO-siRNA)	Saos-2	60	70%	p < 0.001
MTT (Anticancer, GO-siRNA)	Saos-2	80	60%	p < 0.001
MTT (Anticancer, GO-siRNA)	Saos-2	100	50%	p < 0.001
MTT (Anticancer, GO-siRNA)	MG-63	0 (Control)	100%	-
MTT (Anticancer, GO-siRNA)	MG-63	20	85%	p < 0.05
MTT (Anticancer, GO-siRNA)	MG-63	40	75%	p < 0.01
MTT (Anticancer, GO-siRNA)	MG-63	60	65%	p < 0.001
MTT (Anticancer, GO-siRNA)	MG-63	80	55%	p < 0.001
MTT (Anticancer, GO-siRNA)	MG-63	100	45%	p < 0.001

Table S2: Quantitative Values of the qPCR Results

Cytokine	Dose (10 mg/mL GO)	Time Point (day)	Fold Change (vs. Day 0 Control)	p-value (vs. Control)
IL-6	0.5	0	1.0	-
IL-6	0.5	7	4.0	p < 0.01
IL-6	0.5	14	3.0	p < 0.01
IL-6	0.5	21	1.5	p < 0.05
IL-6	0.5	28	1.0	p > 0.05
IL-6	1.0	0	1.0	-
IL-6	1.0	7	3.5	p < 0.01
IL-6	1.0	14	2.8	p < 0.01
IL-6	1.0	21	1.8	p < 0.05
IL-6	1.0	28	1.2	p > 0.05
TNF-α	0.5	0	1.0	-
TNF-α	0.5	7	3.8	p < 0.01
TNF-α	0.5	14	2.9	p < 0.01
TNF-α	0.5	21	1.6	p < 0.05
TNF-α	0.5	28	1.1	p > 0.05
TNF-α	1.0	0	1.0	-
TNF-α	1.0	7	3.2	p < 0.01
TNF-α	1.0	14	2.5	p < 0.01

TNF-α	1.0	21	1.7	$p < 0.05$
TNF-α	1.0	28	1.3	$p > 0.05$
TGF-β	0.5	0	1.0	-
TGF-β	0.5	7	3.5	$p < 0.01$
TGF-β	0.5	14	2.7	$p < 0.01$
TGF-β	0.5	21	2.0	$p < 0.05$
TGF-β	0.5	28	1.4	$p > 0.05$
TGF-β	1.0	0	1.0	-
TGF-β	1.0	7	3.0	$p < 0.01$
TGF-β	1.0	14	2.3	$p < 0.01$
TGF-β	1.0	21	1.9	$p < 0.05$
TGF-β	1.0	28	1.5	$p > 0.05$
IL-1α	0.5	0	1.0	-
IL-1α	0.5	7	4.0	$p < 0.01$
IL-1α	0.5	14	3.2	$p < 0.01$
IL-1α	0.5	21	1.4	$p < 0.05$
IL-1α	0.5	28	1.0	$p > 0.05$
IL-1α	1.0	0	1.0	-
IL-1α	1.0	7	3.7	$p < 0.01$
IL-1α	1.0	14	2.9	$p < 0.01$
IL-1α	1.0	21	1.6	$p < 0.05$
IL-1α	1.0	28	1.2	$p > 0.05$
IL-10	0.5	0	1.0	-
IL-10	0.5	7	4.0	$p < 0.01$
IL-10	0.5	14	3.1	$p < 0.01$
IL-10	0.5	21	1.3	$p < 0.05$
IL-10	0.5	28	1.0	$p > 0.05$
IL-10	1.0	0	1.0	-
IL-10	1.0	7	3.6	$p < 0.01$
IL-10	1.0	14	2.8	$p < 0.01$
IL-10	1.0	21	1.5	$p < 0.05$
IL-10	1.0	28	1.1	$p > 0.05$
MIP-1β	0.5	0	1.0	-
MIP-1β	0.5	7	4.0	$p < 0.01$
MIP-1β	0.5	14	3.0	$p < 0.01$
MIP-1β	0.5	21	1.5	$p < 0.05$
MIP-1β	0.5	28	1.0	$p > 0.05$
MIP-1β	1.0	0	1.0	-
MIP-1β	1.0	7	3.4	$p < 0.01$
MIP-1β	1.0	14	2.6	$p < 0.01$
MIP-1β	1.0	21	1.7	$p < 0.05$
MIP-1β	1.0	28	1.3	$p > 0.05$

Western Blot Images

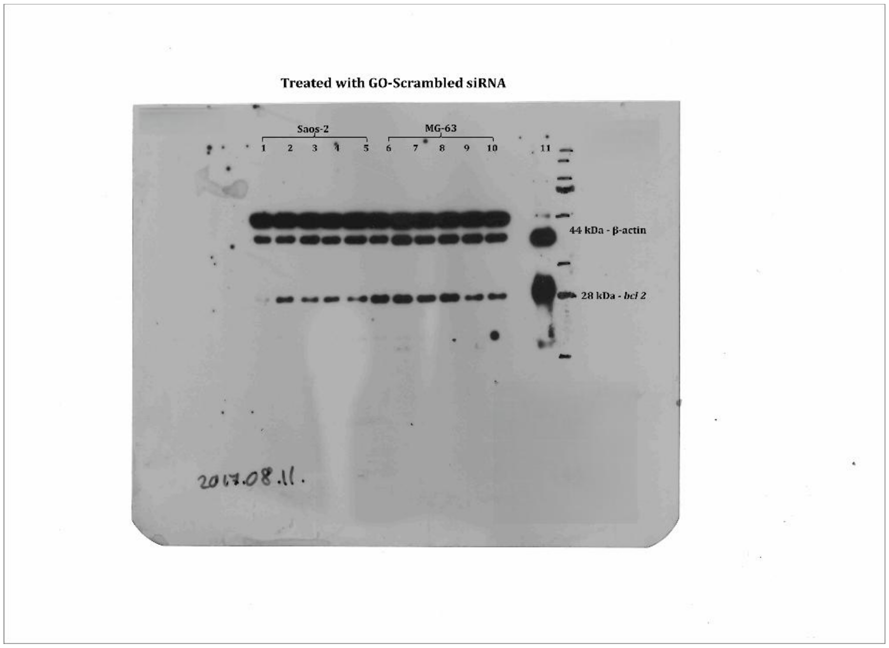

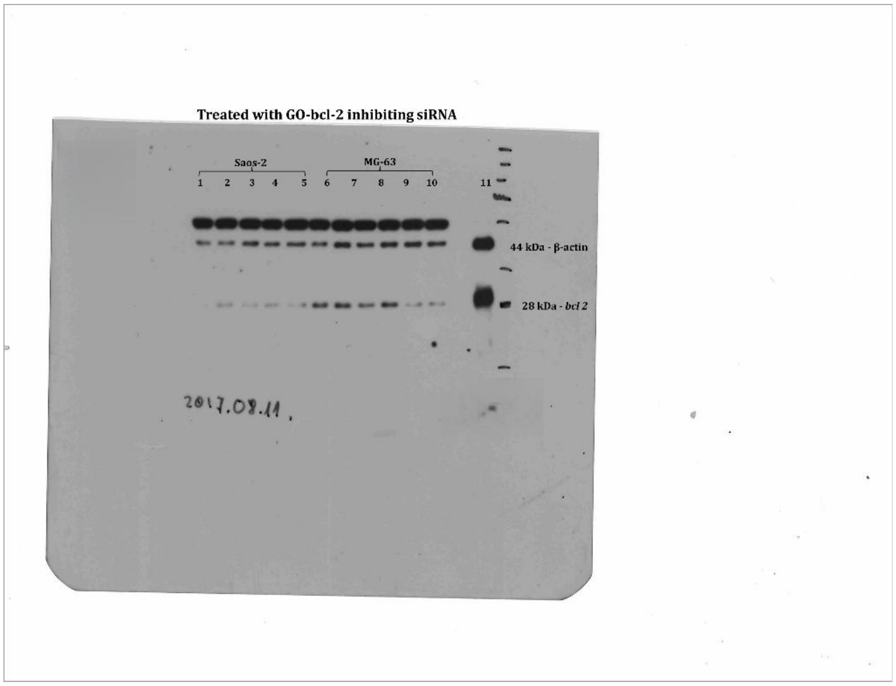

Figure S1 Unprocessed Western blot analysis of bcl-2 knockdown efficiency mediated by GO-siRNA complexes in Saos-2 (lanes 1–5) and MG-63 (lanes 6–10) cells. β-actin (44 kDa) served as loading control; bcl-2 (28 kDa) was probed in both scrambled siRNA-treated groups and bcl-2-inhibiting siRNA-treated groups. Lanes represent increasing GO-siRNA volumes in the range of 20 μL, 40 μL, 60 μL, 80 μL, and 100 μL (from Lanes 1-5 & Lanes 6-10).
